# Right Upper Quadrant Pain: A Rare Presentation of Diverticulitis

**DOI:** 10.7759/cureus.55674

**Published:** 2024-03-06

**Authors:** Rediet Tefera Atalay, Oluwapelumi Kolawole, Girma M Ayele, Abay A Gobezie, Angesom Kibreab, Miriam B Michael

**Affiliations:** 1 Internal Medicine, Howard University Hospital, Washington DC, USA; 2 Internal Medicine, Howard University College of Medicine, Washington DC, USA; 3 Gastroenterology, Howard University Hospital, Washington DC, USA; 4 Internal Medicine, Howard University Hospital, Washingon DC, USA; 5 Internal Medicine, University of Maryland, Baltimore, USA

**Keywords:** appendicitis, right-sided diverticulitis, diverticula, diverticulitis, right upper quadrant pain

## Abstract

Acute diverticulitis is a prevalent medical condition with increasing incidence rates. While the sigmoid colon is the most commonly affected part of the large intestine, there have been occurrences of right-sided diverticulitis although uncommon. We present a case report highlighting the atypical presentation of diverticulitis in a 27-year-old female patient. The patient experienced right upper and epigastric pain and was ultimately diagnosed with right-sided diverticulitis, supported by her CT imaging findings. This uncommon presentation underscores the importance of considering diverse clinical manifestations when diagnosing and managing diverticulitis.

## Introduction

Diverticular disease is more prevalent in older individuals within Western populations, affecting nearly 65% of the population by the age of 85 [1].The prevalence of diverticulitis is greater among men under the age of 50, but it becomes increasingly apparent among older women. Hospitalization rates were higher for men under 50, whereas they were elevated for women over 50 in multiple hospital review datasets. Studies indicate that diverticulosis is found in less than 10% of individuals under the age of 40 [2,3]. In most cases, patients remain asymptomatic throughout their lives, with the diagnosis often made incidentally during endoscopy or cross-sectional imaging [4]. Various factors, including advancing age, low dietary fiber intake, reduced physical activity, obesity, smoking, and heavy alcohol consumption, elevate the risk of developing acute diverticulitis [5]. The severity of diverticulitis varies widely, from mild abdominal pain to the more severe development of an acute abdomen due to perforation and peritonitis [6]. In Western countries, right-sided diverticulitis is uncommon, representing only 1.5% of recorded cases [7]. This case report details a rare occurrence of right-sided diverticulitis in a 27-year-old African American female.

## Case presentation

**Figure 1:**
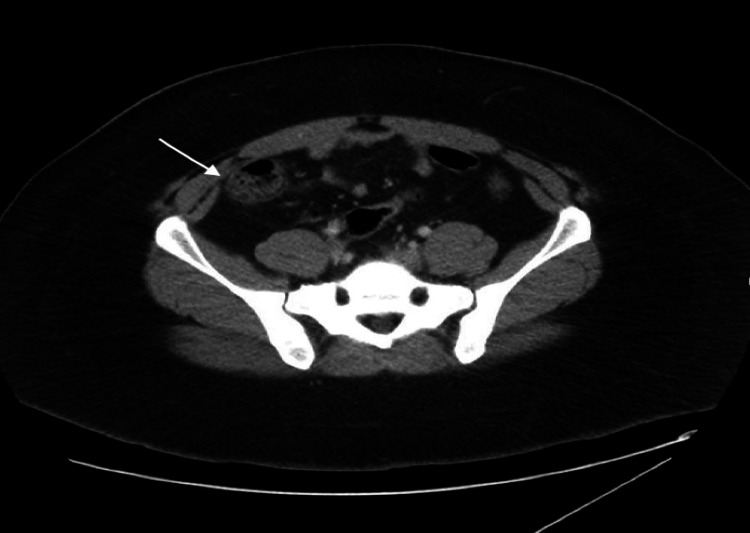
CT abdomen - axial view The arrow points towards the diverticula.

**Figure 2:**
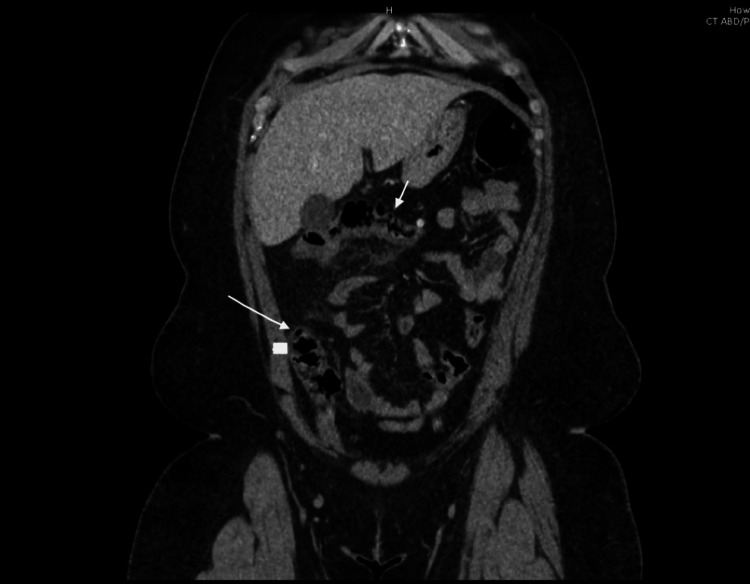
CT abdomen - coronal view CT of the abdomen with contrast showing diverticulosis in the transverse colon, hepatic flexure, and thickening of the hepatic flexure wall, accompanied by pericolonic fat stranding. The arrow points towards the diverticula.

A 27-year-old African American female with an unremarkable medical and surgical history presented to the Emergency Department with acute abdominal discomfort localized in the epigastric and right upper quadrant regions, originating two days before her visit. She described the pain as severe, with a 10/10 sharp sensation that extended to her back. Concurrently, she reported experiencing symptoms such as nausea, reduced appetite, headache, chills, and night sweats over the same two-day period. Although the patient did not subjectively report fever, her temperature upon presentation was 39.1^o^C. Other vital signs were stable and on physical examination, there was epigastric and right upper quadrant tenderness. Laboratory results showed leukocytosis of 21,460 with left shift. The urine pregnancy test was negative, and lipase, kidney, and liver function tests were normal. Ultrasound (US) of the abdomen and pelvis showed a benign left ovarian cyst. CT abdomen pelvis w/ contrast showed ascending colon/transverse colon/hepatic flexure diverticulosis with hepatic flexure wall thickening with pericolic fat stranding suggestive of actue diverticulitis. Figures 1 and 2 show the CT scan of the abdomen with axial and coronal view, respectively.

Our patient initially received IV ceftriaxone and metronidazole, fluids, and analgesics. Antibiotics were later switched to oral ciprofloxacin and metronidazole. The patient has been scheduled for an outpatient colonoscopy in six weeks and advised to follow up with colorectal surgery, as a surgical intervention may be necessary in the long term. 

## Discussion

While left-sided diverticulitis is prevalent in Western populations, right-sided diverticulitis is considered uncommon [8]. The Asian population, however, exhibits a higher incidence of right-sided diverticulitis, with genetic factors and diet presumed to influence its onset [1]. The rising occurrence of diverticular disease in other regions of the world may be attributed to the adoption of Western lifestyle and dietary habits [1]. US and CT scans play a crucial role in distinguishing diverticulitis from acute appendicitis, especially since relying solely on history and physical examination can be challenging [9]. Although right-sided diverticulitis tends to be less severe and predominantly occurs in young males compared to its left-sided counterpart, which is associated with worse laboratory findings such as elevated C-reactive protein (CRP), creatinine, and low hemoglobin and albumin [10].

CT scanning is considered the superior imaging modality for diagnosing possible acute right-sided colonic diverticulitis (ARCD) [11]. However, US is a more economical and radiation-free alternative, which is particularly relevant for younger patients with right-sided diverticulitis. US features can offer valuable preoperative diagnostic information, such as diverticular wall thickening, surrounding echogenic fat, and intra-diverticular echogenic material [12,13]. While US demonstrates good diagnostic accuracy for diverticulitis compared to CT scans, it is less effective in assessing the extent of large abscesses and detecting free air. Moreover, its accuracy is compromised in obese patients and those with obstructing gas. However, US remains a preferable option for pregnant women to avoid exposure to ionizing radiation [14]. However, the quality of US results relies on the operator's expertise. In cases where US findings are inconclusive, a contrast-enhanced CT scan can be a useful complementary tool [12,13].

The diagnostic and treatment principles for ARCD closely mirror those for acute left-sided colonic diverticulitis (ALCD). Distinct management guidelines are lacking for ARCD. Non-operative approaches are typically favored, especially in cases without widespread peritonitis, although distinguishing between benign and malignant instances before surgery can pose challenges [15]. The utilization of antibiotic medication for treating uncomplicated acute diverticulitis is deemed crucial, though its use remains a subject of controversy, with selective administration recommended for patients with uncomplicated acute diverticulitis [9]. Surgical intervention is commonly reserved for complicated cases [16]. 

In a study by Zhin Chen et al. on 111 cases of cecal and ascending colonic diverticulitis, 13 patients underwent resection and repair for right-sided colonic diverticulitis, while no patients had colectomy. Also, 15 patients with right-sided colonic diverticulitis underwent abdominal drainage, and the remainder were managed nonoperatively. The length of hospital stay did not significantly differ among the three treatments for right-sided colonic diverticulitis [17].

A review of observational cohort studies on nonoperative management of right diverticulitis that included 1,584 adult participants from 11 studies to evaluate the primary outcomes of the recurrence rate and morbidity associated with recurrence was done. The pooled recurrence rate over a median follow-up of 34.2 months was 12% (95% CI, 10%-15%). Only 9.9% of patients required urgent surgery at the first recurrence, and there were no reported mortalities [16]. The study concluded that non-operative management in right-side diverticulitis is safe, and the risk of recurrence is rare [16].

A recent meta-analysis encompassing 38 studies and involving 10,129 patients examined the outcomes of non-operative management. The analysis revealed low rates of recurrence and treatment failure for both right-sided and left-sided Crohn's disease (CD), with left-sided CD showing a slightly higher recurrence rate. Interestingly, the recurrence rates were similar regardless of whether patients received antibiotics for uncomplicated CD or not [18]. 

## Conclusions

Diverticulitis traditionally manifests with left lower quadrant pain and is primarily diagnosed in the elderly population. According to available literature, right-sided diverticulitis is rare and appears to occur more frequently among Asian populations. Its manifestation with symptoms such as abdominal pain in the right lower quadrant, tenderness, nausea, or vomiting may sometimes be mistaken for acute appendicitis, further complicating diagnosis. We emphasize the significance of considering diverticulitis as a potential diagnosis in atypical cases, especially in younger populations. Studies indicate that once the diagnosis is established, there are no distinct management guidelines for right-sided diverticulitis, and the management approach is similar to that of left-sided diverticulitis.
